# Supplementary material for: Hematin- and Hemin-Induced Spherization and Hemolysis of Human Erythrocytes Are Independent of Extracellular Calcium Concentration
Source: Cells. 2024 Mar 21;13(6):554. doi: 10.3390/cells13060554 (PMC10969559; doi:10.3390/cells13060554)
Supplement: Supplementary file 1 [file cells-13-00554-s001.zip › cells-2892772-supplementary.pdf]

SUPPLEMENTARY MATERIALS

S1. Hemin and hematin

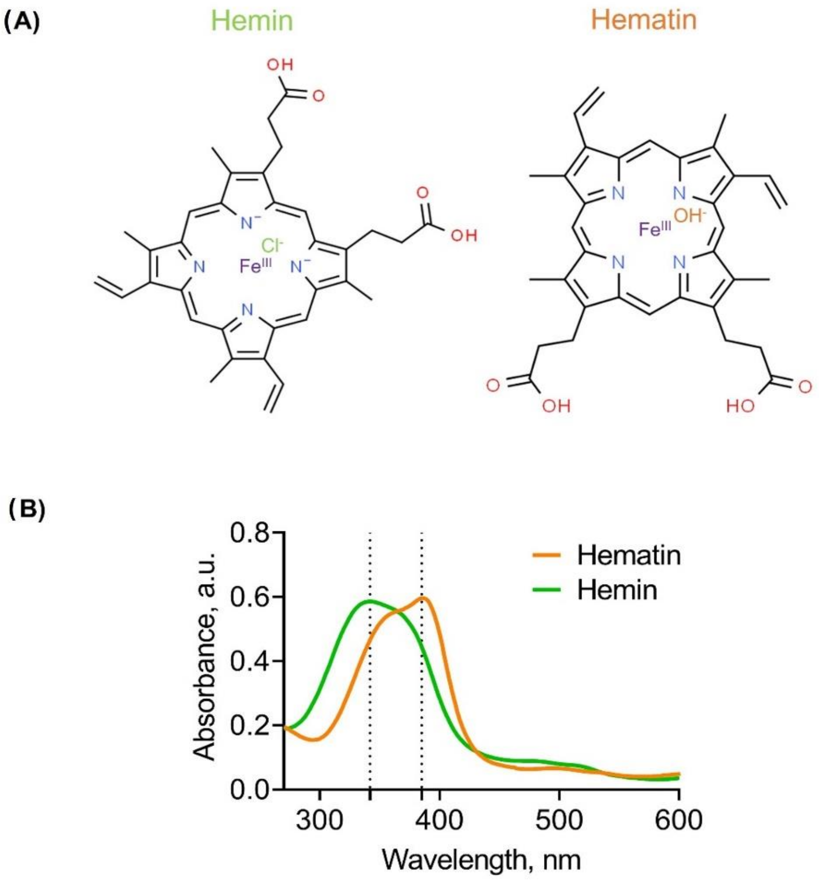

Supplementary Figure S1. Hematin and hemin characterization. (A) Structural formulas of hematin and hemin. Hematin and hemin are oxidized heme derivatives, which have tetrapyrrole macrocycle linked together by methylene bridges. In the center there is a Fe^{3+} with coordinating ion: hydroxide group (-OH) in hematin and clorine (-Cl) in hemin **[10].** (B) Hematin and hemin spectra. Hemin powder was dissolved in 20mM NaOH for hematin stock solution and in DMSO for hemin stock solution. Hematin and hemin concentrations were controlled using the molar extinction method according to the Beer-Lambert equation using the compound's millimolar extinction coefficient at two different wavelengths (dotted lines). For hematin $\varepsilon = 58.4$ $mM^{-1}cm^{-1}$ at $\lambda = 385$ nm **[7].** It should be mentioned that in the paper that we quoted here **[7]** hematin is indicated as hemin. Similarly, with spectral data we determined ε for hemin at wavelength $\lambda = 342$ nm and ε was 59.5 $mM^{-1}cm^{-1}$.

S2. Hematin- and hemin-induced release of hemoglobin (Hb)

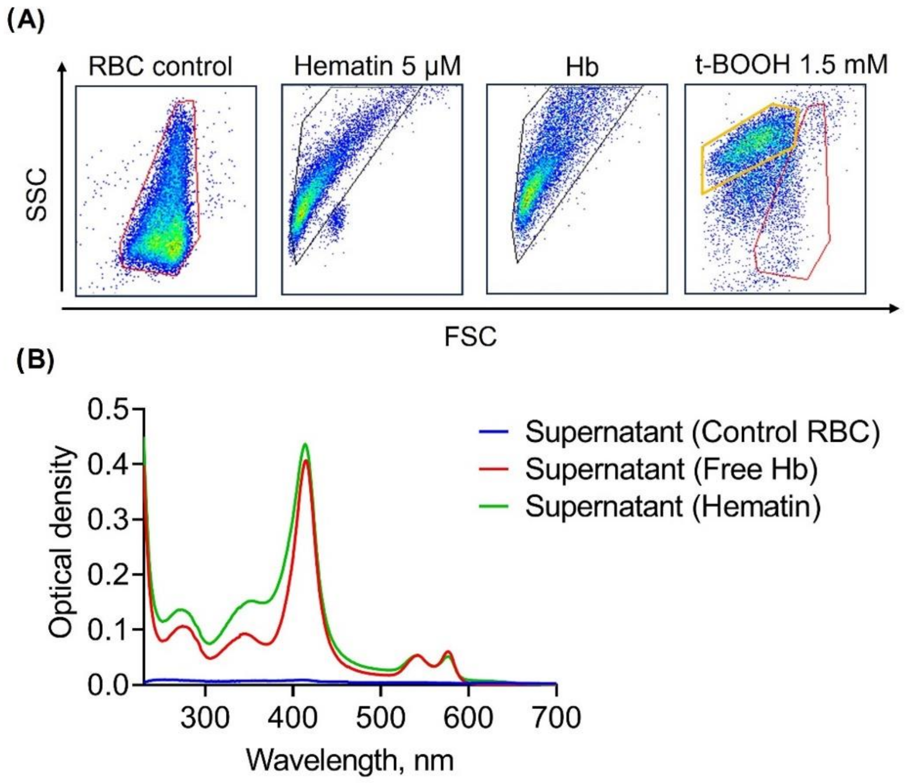

Supplementary Figure S2. Hematin induced release of Hb out of RBCs. To induce RBCs lysis cells were incubated with hematin (5 μM, 5 min), t-BOOH (1.5 mM, 3h), as a control for microparticle formation (yellow gate) and analyzed by flow cytometry. Red gate corresponded to control RBCs, black gate – RBCs lysate after incubation with hematin (A). RBCs were centrifuged at 400g. Collected supernatants were additionally centrifuged at 20,000g for 70 minutes to separate free Hb and pellet-containing possible MPs. Then supernatants were characterized by spectrophotometry (B).

S3. Light scattering indicatrix of human RBCs

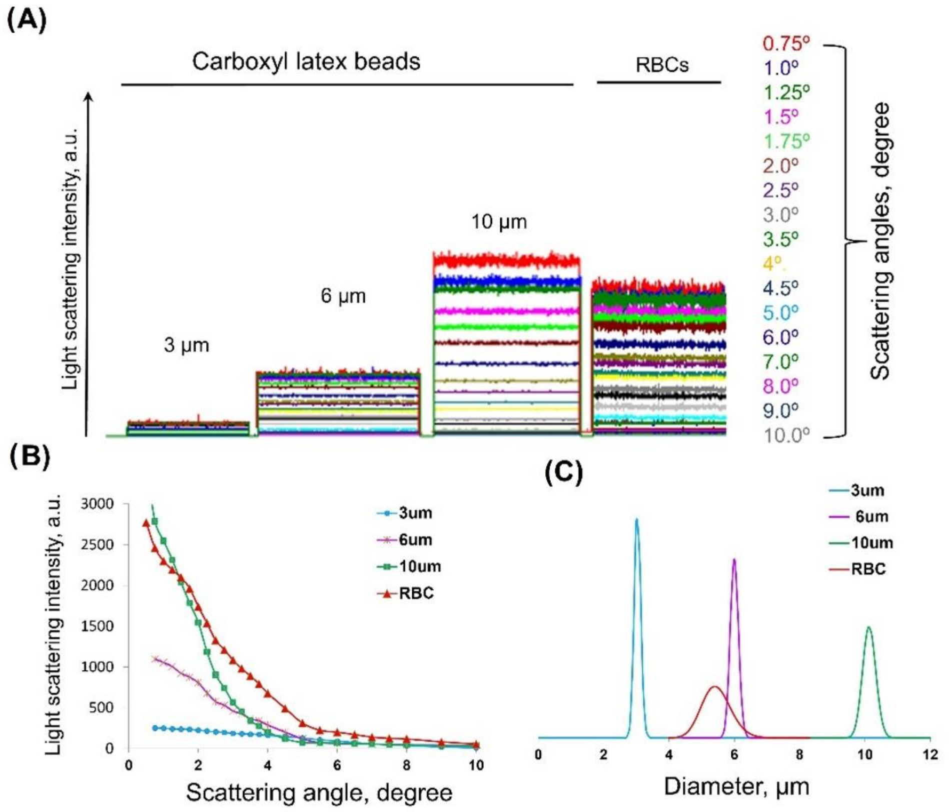

Supplementary Figure S3. Development of the approach for absolute cell volume estimation. (A) – carboxyl latex beads, or washed RBCs (10^6 cells/mL) were suspended as indicated in HEPES buffer and the corresponding light scattered intensity was registered; (B) – the light scattering intensities from S2A were plotted against the scattering angles to obtain the corresponding scattering indicatrix; (C) – in accordance with the ratio of indicatrices for the analyzed carboxyl beads, and RBCs were plotted on a scale for the diameters.

S4. Analysis of RBCs spherization by laser diffraction

LaSca-TM laser analyzer registers light scattering intensity (LSI) at the scattering angles from 1° up to 12°. In our experiments we used only 2.5° and 12° due to high sensitivity to hematin/hemin-induced RBCs transformation. The more detailed explanation of this method is described in **[29, 32].** Previously it was shown that LaSca-TM laser particle analyzer can be used for platelet shape change registration. We adapted this method for RBCs and introduced parameters for RBCs transformation: Spherization of RBCs corresponded to the decrease of LSI noise level in contrast to control. To characterize this process, we introduced special parameters: spherization index and rate (V) of spherization. V spherization is a parameter determined as the rate of LSI increase measured at 12°. Spherization index is ratio of control LSI amplitude ($\sigma^2_{control}$) to LSI after hematin/hemin addition ($\sigma^2_{spherization}$). The parameter σ^2 and V spherization was calculated by the original software LaSca_32 v.1498 (BioMedSystems Ltd., Saint Petersburg, Russia).

Spherization index (%) was calculated as follows:

$$\textbf{Spherization index} = 100 - \sigma^2_{spherization} / \sigma^2_{control} \times 100\ \% \qquad (1)$$

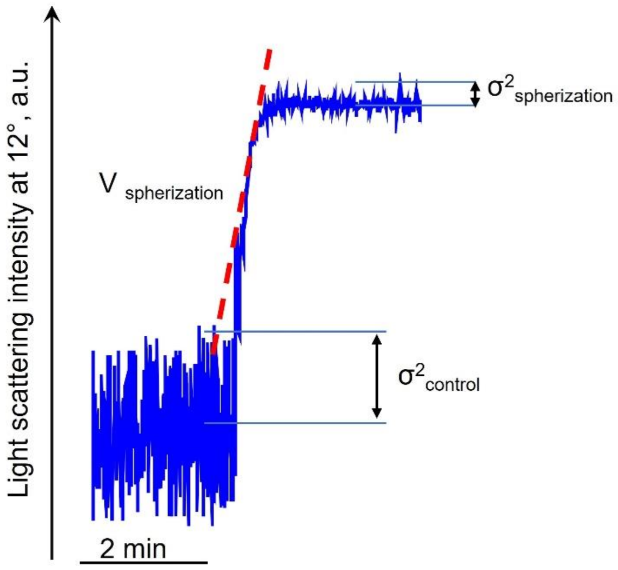

Supplementary Figure S4. Calculation of the RBCs spherization index by laser diffraction method. RBCs diluted in the HEPES buffer (12 µL, 10^6 cells/mL final concentration) were added to the cuvette with a continuous stirring at 37 °C to register light scattering intensity corresponding to control. Then hematin or hemin were added to the cells in cuvette in the range of concentration 5-700 nM and 5-1500 nM respectively. Oscillation contracting represents spherization of RBCs.

S5. RBC lysis characterization by laser diffraction

Hemolysis of RBCs corresponds to the decrease of LSI signal in all registered angles. To characterize hemolysis, we calculated precent of hemolysis (% hemolysis) and rate (V) of hemolysis. % hemolysis is the ratio of minimum (**I_{hem}**) LSI signal to maximum (**I_0**). V hemolysis corresponds to the rate of LSI decrease measured at 12°.
Hemolysis % was calculated as follows:

$$\textbf{Hemolysis\%} = (1 - I_0/I_h) \times 100\% \qquad (2)$$

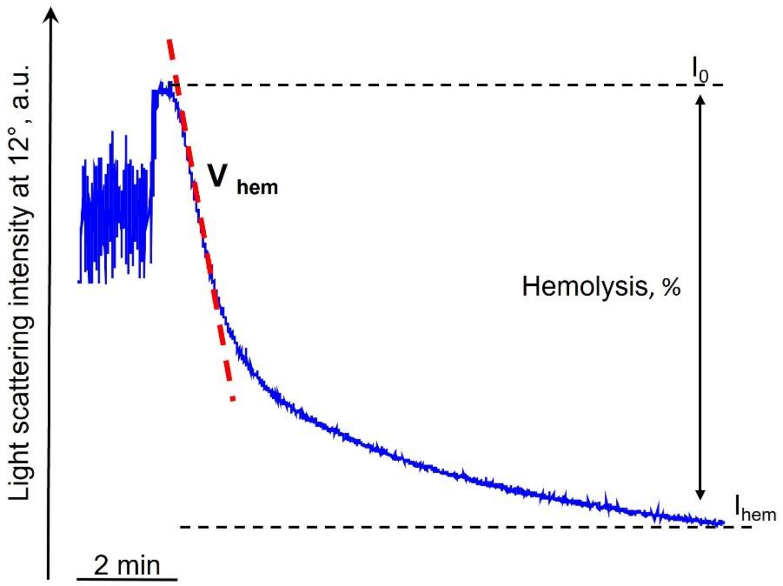

Supplementary Figure S5. Calculation of RBCs Hemolysis % by laser diffraction method. RBCs diluted in the HEPES buffer (10 μL, 10^6 cells/mL final concentration) were added to the cuvette with a continuous stirring at 37 °C to register light scattering intensity corresponding to control. Then hemin/hematin were added to the cells in the cuvette. Hemolysis of RBCs started in excess of hemin/hematin inducing spherization. Signal reduction of light scattering intensity in all angles of scattering corresponded to hemolysis of RBCs. Data obtained by Laser diffraction method was analyzed by the original software LaSca_32 v.1498 (BioMedSystems Ltd., Saint Petersburg, Russia) of the laser particle analyzer LaSca-TM.

S6. Analysis of $[Ca^{2+}]_i$ changes using LaSca-TMF

The detailed description for calibration intracellular calcium concentration in platelets is shown in **[44].** We adapted this method for RBCs. Briefly, we used A23187 to achieve maximum ($F_{max)}$ of fluorescence signal of Fluo-3-stained RBCs and EGTA for minimum (F_{min}). F is the fluorescence at intermediate calcium levels. The initial [Ca^{2+}]i was estimated to be around 50 nM which corresponds to physiological intracellular calcium level in RBCs (1).

To calculate $[Ca^{2+}]_i$ Equation 3 was used:

$$[Ca^{2+}]_i = K_d(F - F_{min})/(F_{max} - F) \qquad (3)$$

where K_d is a dissociation constant for Ca^{2+} which is reported (according to the manufacturer) to be 390 nM;

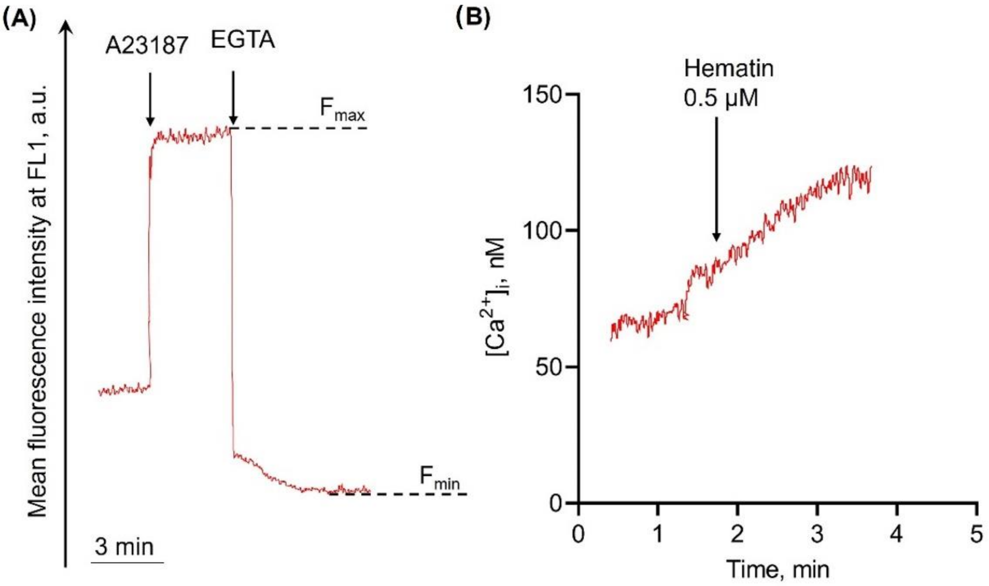

Supplementary Figure S6. Calibration of $[Ca^{2+}]_I$ in RBCs. RBCs after loading with Fluo-3-AM (10 μM, 1 hour, 37°C) were diluted in HEPES buffer (10^6 cells/mL final concentration) in the cuvette with a continuous stirring (1200 rpm) at 37ºC and registered as a control. Then calcium ionophore (A23187) was added to the cells and maximal fluorescence (Fl) signal (F_{max}) was achieved. After that EGTA (16 mM) was added to bound the Ca2+ ions and we registered minimal Fl signal (F_{min}). F is the fluorescence at intermediate calcium levels. (A) Representative curve of Fl signal of Fluo-3 measured at 529 nm (LaSca-TMF analyzer). (B) Hematin-induced $[Ca^{2+}]_I$ change registered in dynamics and calculated according to equation 3.

S7. Lactadherin synthesis

Expression vector construction. The coding sequence of bovine lactadherin C2 domain (LactC2) and codon optimized mNeonGreen were obtained from Lact-C2-GFP plasmid **[34]** (Lact-C2-GFP was a gift from Sergio Grinstein (Addgene plasmid # 22852; http://n2t.net/addgene:22852; RRID:Addgene_22852) and pmNeonGreenHO-G plasmid (2) (pmNeonGreenHO-G was a gift from Isei Tanida (Addgene plasmid # 127912; http://n2t.net/addgene:127912; RRID:Addgene_127912). PCR was used to amplify LactC2 and mNeonGreen open reading frames using Q5 High-Fidelity Polymerase (NEB) according to the manufacturer instructions. Restriction sites were introduced as 5′ primer overhangs. Subsequently, LactC2 and mNeonGreen-encoding fragments were subcloned into pET-28a(+) (Novagen Darmstadt, Germany) vector using T4 ligase (NEB, MA, USA). Resulting protein sequence has a (GGGGS)3RSRAQAL linker between N-terminal mNeonGreen and LactC2. Obtained plasmids were propagated in DH5a E.Coli cells. Plasmid clones were isolated using QIAprep Spin Miniprep Kit (Qiagen, Hilden, Germany). Correct final sequence was confirmed by Sanger sequencing using standard T7 forward and reverse primers.

Protein Synthesis and Purification. E. coli strain Rosetta-Gami 2 (DE3) pLysS was transformed with pET28a containing mNeonGreen-LactC2 fusion sequence and glycerol stocks were prepared **[27].** For protein synthesis 100 ml of autoinduction 2xYT containing 0.5% v/v glycerol and 100 µg/ml kanamycin was inoculated with 20 µl of the glycerol stock and outgrown for 4 h at 37°C followed by 36 h at 20°C in an orbital shaker at 250 RPM. Bacteria were harvested by centrifugation at 5000xg for 10 min. Obtained pellet was lysed with B-PER™ Complete (Thermo Scientific, MA, USA) and additionally disrupted by sonication. Crude lysate was clarified by centrifugation at 8000xg at 4°C for 30 min. Protein isolation was performed using gravity columns packed with 1 mL of Ni-NTA resin (Qiagen Hilden, Germany), according to the manufacturer instructions. The protein was eluted into three fractions 1 mL each. Ni-NTA chromatography results were confirmed by SDS-PAGE. Protein containing fractions were pooled and buffer exchanged using 3kDa Amicon® Ultra 15 mL Centrifugal Filter (Merck, Darmstadt, Germany) into a storage buffer containing 20 mM HEPES, 150 mM NaCl, 50 mM trehalose, pH 7.4.

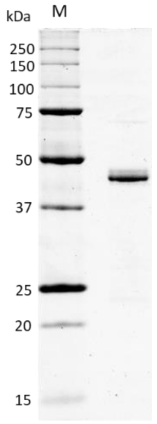

Supplementary Figure S7. Coumassie-stained SDS-PAGE containing buffer-exchanged recombinant mNeonGreen-lactadherin C2 domain fusion protein. mNG-lactC2; 400 ng protein was loaded in 10% SDS-PAGE gel. M shows protein molecular weight marker. Imaging was performed with ChemiDoc MP Imaging system using Epi Far-Red illumination (BioRad, CA, USA).
